# A multicopy Y-chromosomal SGNH hydrolase gene expressed in the testis of the platyfish has been captured and mobilized by a *Helitron* transposon

**DOI:** 10.1186/1471-2156-15-44

**Published:** 2014-04-08

**Authors:** Marta Tomaszkiewicz, Domitille Chalopin, Manfred Schartl, Delphine Galiana, Jean-Nicolas Volff

**Affiliations:** 1 Institut de Génomique Fonctionnelle de Lyon, Ecole Normale Supérieure de Lyon, Centre National de la Recherche Scientifique UMR 5242, Université de Lyon I, 46 allée d'Italie, Lyon 69364, Lyon Cedex 07, France; 2 Physiologische Chemie, Biozentrum, University of Würzburg, Am Hubland, and Comprehensive Cancer Center, University Clinic Würzburg, Josef Schneider Straße 6, Würzburg 97074, Germany; 3 Present address: Center for Medical Genomics, Department of Biology, Penn State University, University Park, PA 16802, USA

**Keywords:** Platyfish, *Xiphophorus maculatus*, Sex determination, Sex chromosomes, Y chromosome, Testis, SGNH hydrolase, *Helitron*, Transposition

## Abstract

**Background:**

Teleost fish present a high diversity of sex determination systems, with possible frequent evolutionary turnover of sex chromosomes and sex-determining genes. In order to identify genes involved in male sex determination and differentiation in the platyfish *Xiphophorus maculatus*, bacterial artificial chromosome contigs from the sex-determining region differentiating the Y from the X chromosome have been assembled and analyzed.

**Results:**

A novel three-copy gene called *teximY* (for testis-expressed in *Xiphophorus maculatus* on the Y) was identified on the Y but not on the X chromosome. A highly related sequence called *texim1*, probably at the origin of the Y-linked genes, as well as three more divergent *texim* genes were detected in (pseudo)autosomal regions of the platyfish genome. *Texim* genes, for which no functional data are available so far in any organism, encode predicted esterases/lipases with a SGNH hydrolase domain. Texim proteins are related to proteins from very different origins, including proteins encoded by animal CR1 retrotransposons, animal platelet-activating factor acetylhydrolases (PAFah) and bacterial hydrolases. *Texim* gene distribution is patchy in animals. *Texim* sequences were detected in several fish species including killifish, medaka, pufferfish, sea bass, cod and gar, but not in zebrafish. *Texim*-like genes are also present in *Oikopleura* (urochordate), Amphioxus (cephalochordate) and sea urchin (echinoderm) but absent from mammals and other tetrapods. Interestingly, *texim* genes are associated with a Helitron transposon in different fish species but not in urochordates, cephalochordates and echinoderms, suggesting capture and mobilization of an ancestral *texim* gene in the bony fish lineage. RT-qPCR analyses showed that Y-linked *teximY* genes are preferentially expressed in testis, with expression at late stages of spermatogenesis (late spermatids and spermatozeugmata).

**Conclusions:**

These observations suggest either that TeximY proteins play a role in *Helitron* transposition in the male germ line in fish, or that *texim* genes are spermatogenesis genes mobilized and spread by transposable elements in fish genomes.

## Background

In contrast to the situation observed in mammals and birds, where sex determination systems and master sex-determining genes have been conserved over long periods of evolution, sex determination is hypervariable in teleost fish [1-4]. Related fish species and even populations can have different genetic sex-determining systems, with various impact of environmental factors. Fish sex chromosomes are generally poorly differentiated. They are therefore considered to be evolutionary young and might have emerged independently in different fish lineages. Hence, parallel studies on different fish models are necessary to better understand the evolutionary dynamics of sex determination in fish.

Such studies have demonstrated that different, even closely related fish species can have different master sex-determining genes. The first master sex-determining gene in fish has been identified in the medaka *Oryzias latipes*. This gene, *dmrt1bY*, is a Y-chromosomal duplicate of the autosomal gene *dmrt1*, which encodes a transcription factor with DM domain involved in male development in vertebrates [5,6]. Interestingly, *dmrt1bY* is not present in related species from the same genus, which therefore must possess other master sex-determining genes. This is the case in *Oryzias luzonensis*, where the putative master sex-determining gene is *gsdf* (gonadal soma-derived growth factor), another gene from the sex-determining cascade [7].

More distant species have also different master sex-determining genes. In the Patagonian pejerrey *Odontesthes hatcheri*, a Y-linked duplicate of the *amh* gene, which encodes the anti-Müllerian hormone belonging to the TGF-β superfamily, might drive sex determination [8]. In the rainbow trout *Oncorhynchus mykiss*, the master sex-determining *sdY* is a Y-specific duplicate of the interferon regulatory factor 9 gene [9]. Added to the results obtained in *O. latipes*, these observations underline the importance of gene duplications in the formation of new master sex-determining genes in fish. However, the situation is different in pufferfishes from the *Takifugu* genus. In *Takifugu*, phenotypic sex might be controlled by two alleles of the anti-Müllerian hormone receptor type II gene *amhr2*, which is located on both the X and Y chromosomes. A single nucleotide polymorphism differentiating both alleles affects one amino-acid in the kinase domain of Amhr2. Females are always homozygous for one allele, while males are heterozygous [10]. Hence, hypervariability of sex determination might be associated with frequent evolutionary switch of master sex-determining genes at the top of more conserved downstream components of the sex determination and differentiation cascade, following the principle of “masters change, slaves remain” [11]. Further studies on different fish models are necessary to test the extent of this hypothesis.

Another classical fish model to study sex determination is the platyfish *Xiphophorus maculatus*. This species possesses three different types of sex chromosomes: X, Y and W. Males can be XY or YY, while XX, WX and WY genotypes lead to female development [12]. Using a strain with XY male heterogametic sex determination, the positional cloning of sex-linked genes has been initiated. A bacterial artificial chromosome (BAC) genomic library of XY males has been constructed, and BAC contigs covering the region differentiating the X from the Y chromosome have been assembled and sequenced [13,14]. Very recently, the genome of an XX female has been sequenced [15]. The sex-determining region was found to be prone to DNA rearrangements including deletions, transpositions and duplications [12,16]. We report here the identification of a novel gene called *teximY*, which is found in there copies on the platyfish Y chromosome but absent from the X chromosome. This gene is preferentially expressed in testis, and has been captured and mobilized by a *Helitron* transposon in fish.

## Results

### Three *teximY* genes are clustered in the sex-determining region of the platyfish Y chromosome but are absent from the X chromosome

**Figure 1:**
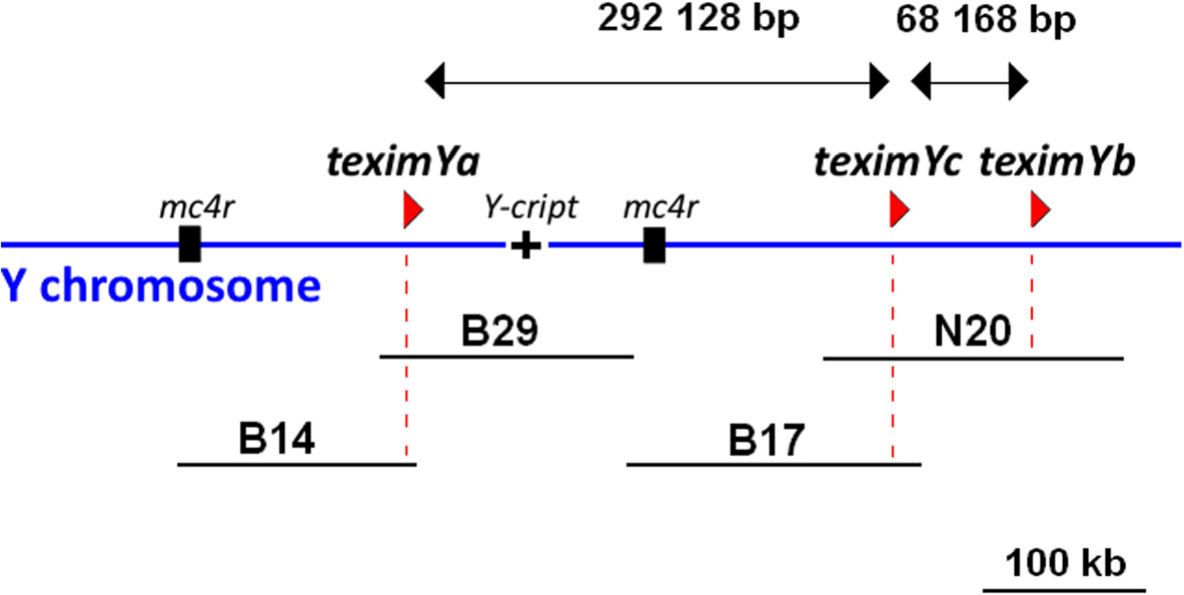
**Structure of the *teximY *gene cluster in the sex-determining region of the Y chromosome of *Xiphophorus maculatus*.** Red arrowheads show the position and predicted transcription orientation of *teximY* copies. The four sequenced BAC clones (B14, B29, B17 and N20) are shown as black lines. The distance between *teximY* copies is given. Locations of the Y-specific *Y-cript* pseudogene and of two copies of the type 4 melanocortin receptor gene *mc4r*[14] are indicated.

Four overlapping Y-linked BAC clones (B14, B29, B17 and N20; Figure 1) from the Rio Jamapa platyfish BAC genome library [13] were sequenced to completion and assembled, resulting in a total sequence of 585,694 bp in length. BAC clone B29 contained a pseudogene called *Y-cript*. PCR analyses demonstrated the presence of *Y-cript* in all males tested (> 200 individuals) but its absence from all females (> 200 individuals) (data not shown). Hence, the four assembled BAC clones are closely linked to the master sex-determining gene on the Y chromosome, or may even contain it.

**Figure 2:**
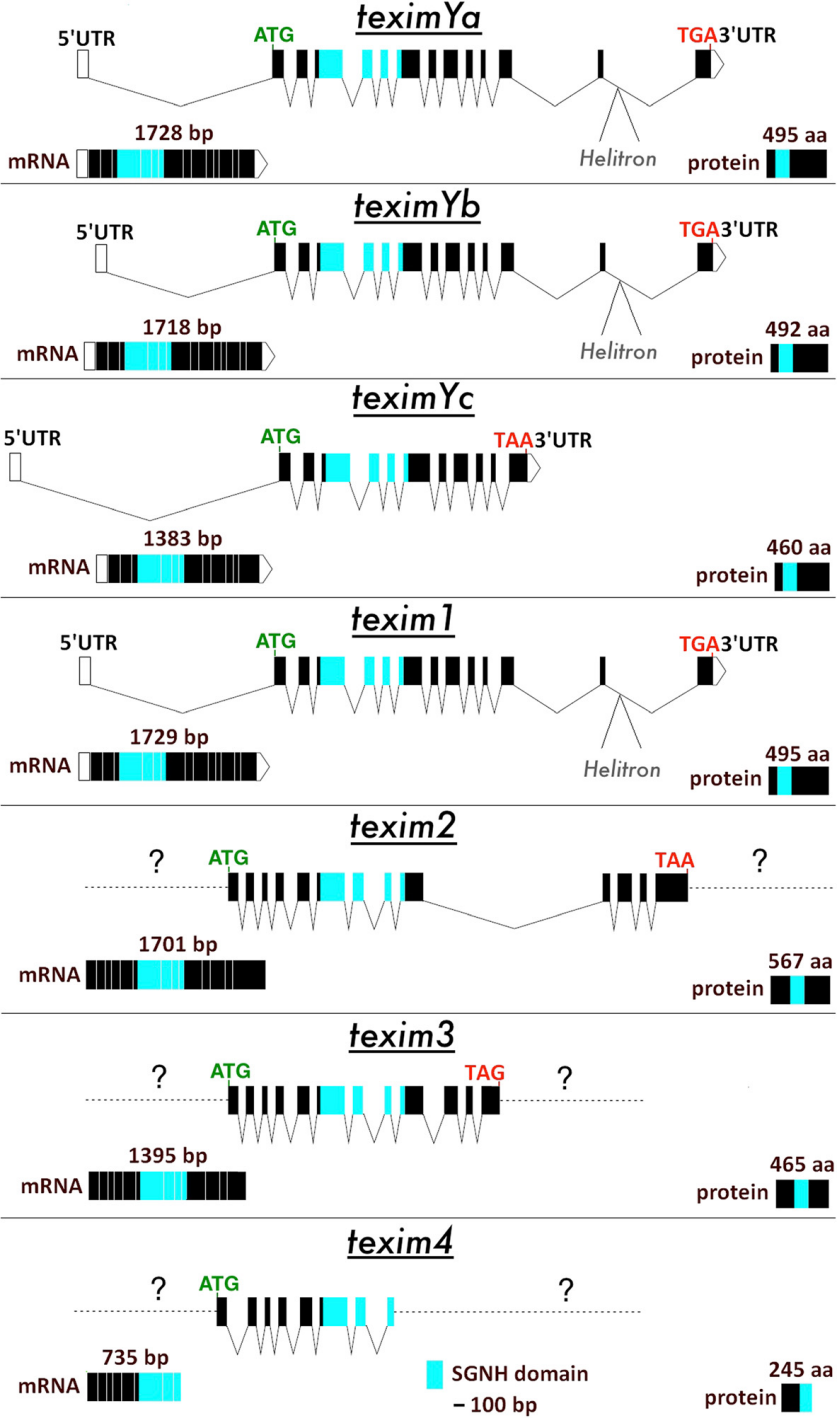
**Exon/intron structure of *texim *genes in the platyfish genome.** The SGNH hydrolase domain(-coding region) is marked in light blue. Introns are represented by broken lines. Predicted start codons are in green and stop codons in red. cDNA and predicted protein sizes are provided. Helitron transposon positions are indicated. Question marks indicate lack of information due to absence of sequence data. Both coding and protein sequences of *texim4* are partial.

Further sequence analysis revealed the presence of three copies of a new gene candidate that was called *teximY* (for testis-expressed in *Xiphophorus maculatus* on the Y) in a region of approximately 300 kb (Figures 1 and 2). These three genes were detected neither in BAC clones specific of the X chromosome nor in the genome sequence of an XX female ([15]; http://www.ensembl.org/Xiphophorus_maculatus/Info/Index). The presence of *teximYa*, *teximYb* and *teximYc* was tested by PCR in 34 platyfish individuals from the Rio Jamapa population (17 males and 17 females). All males tested were positive for the three genes, while all females were negative. This confirmed the presence of *teximYa*, *teximYb* and *teximYc* on the Y chromosome of the platyfish in the Rio Jamapa population. *TeximY* genes are located at distances between 80 and 280 kb near *Y-cript* and linked to two copies of a type 4 melanocortin receptor gene *mc4r*[14].

All three *teximY* copies were in the same transcriptional orientation (Figure 1). They showed 95-98% nucleotide identity at the cDNA level and 89-97% amino acid similarity, suggesting recent duplication events. Software-based gene structure prediction as well as sequence comparison with platyfish expressed sequence tags and sequencing of RT-PCR and RACE-PCR products indicated that the *teximYa* and *teximYb* genes are both composed of 15 exons, while *teximYc* is a truncated version lacking the last two 3′ exons (Figure 2). Sequence comparison of *teximYa* and *teximYb* flanking regions revealed extensive sequence identity for more than 5 kb upstream and more than 7 kb downstream of the genes, suggesting a duplication of at least 25 kb. This analysis could not be performed for *teximYc* neighboring regions due to the missing adjacent genomic sequences.

BLAST analysis of the platyfish female genome sequence [15] revealed the presence of several *teximY-*related sequences. These sequences were not present on sex-chromosomal BAC contigs covering the region differentiating the X from the Y chromosome [13,14]. They are therefore located either on autosomes, or alternatively in homologous regions of the X and the Y chromosomes (pseudoautosomal regions). One copy called *texim1* presented a high level of nucleotide identity (> 95%) with the Y-chromosomal *teximY* genes as well as a very similar gene structure (Figure 2). Hence, *texim1* might correspond to the molecular progenitor of *teximY* sequences, or *texim1* is a (pseudo)autosomal duplicate of one of the *teximY* genes. Three more divergent *texim* genes were also detected in the genome, with one of them, *texim2*, mapping to autosomal linkage group 18. Altogether, four copies of *texim* are present in platyfish (pseudo)autosomal parts of the genome, with three additional Y-specific copies only in males.

### *Texim* genes encode putative SGNH hydrolases found in fish and other metazoans

**Figure 3:**
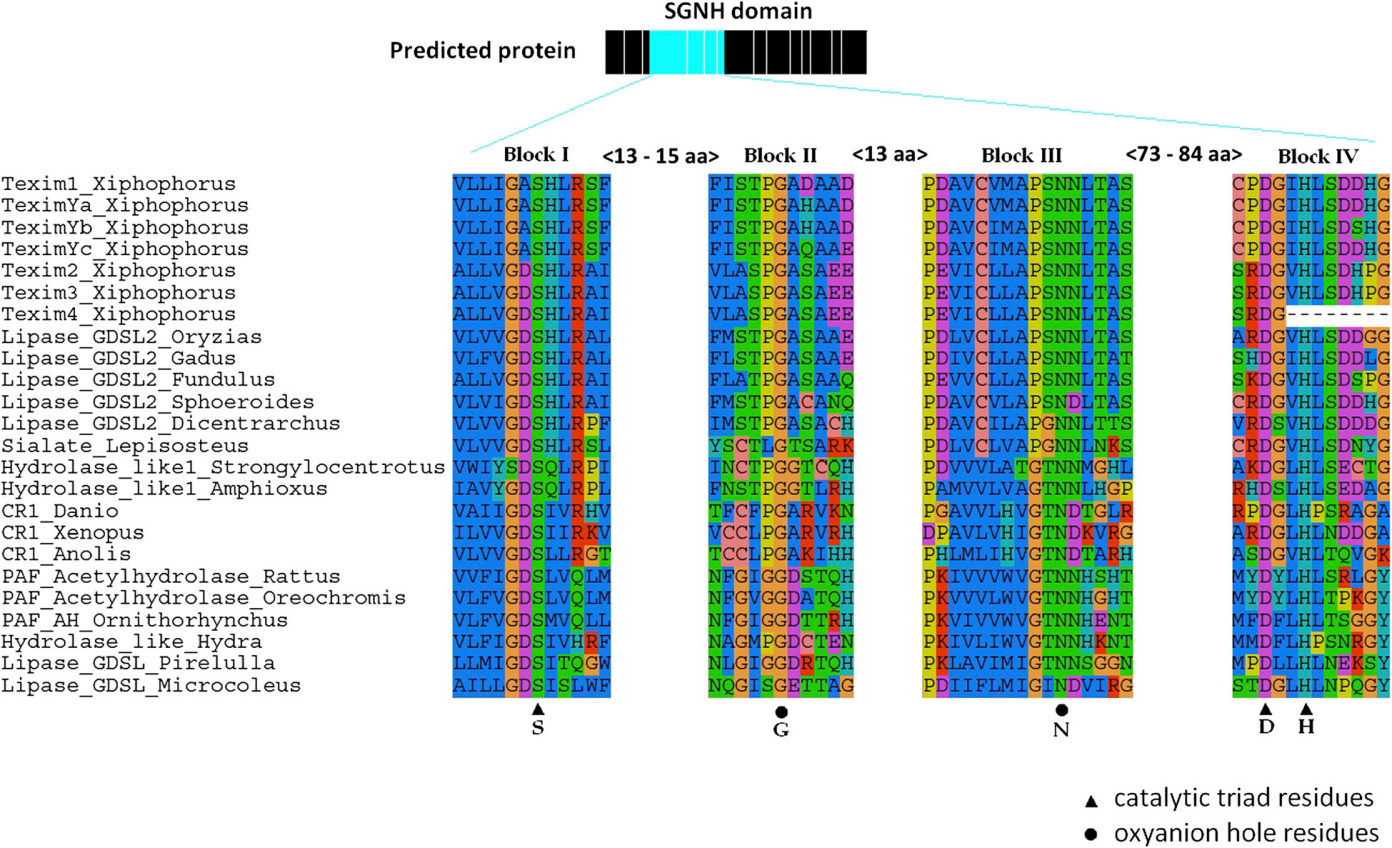
**Conserved amino-acid blocks in the SGNH domain of predicted Texim proteins.** Critical residues important for catalytic activity and oxyanion hole residues are indicated, as well as minimum-maximum amino-acid distances between the domains.

*In silico* analysis of conserved domains identified a SGNH hydrolase domain in the predicted Texim proteins. This domain, which is found in certain esterases and lipases with broad substrate specificity and regiospecificity [17], is characterized by four invariant catalytic residues: the catalytic serine (S) at the N-terminus in conserved block I, a glycine (G) in conserved block II, an asparagine (N) in conserved block III and the catalytic histidine (H) in conserved block IV (Figure 3). An aspartate residue (D) is often present in block IV. The four major residues (S, G, N and H) are important for activity, with a different catalytic mechanism when compared to common alpha/beta-hydrolases [18].

Sequence database analysis identified *texim* sequences in different fish species including killifish, medaka (2 very similar copies on chromosomes 10 and 21), pufferfish (one copy in *Sphoeroides nephelus*), sea bass, Atlantic cod (one copy) and gar (one copy). No sequence was detected in zebrafish. *Texim* sequences were also found in *Oikopleura* (urochordate, one copy), in Amphioxus (cephalochordate, three copies) and sea urchins (echinoderms, two copies). *Texim* genes were absent from mammals and other tetrapods, suggesting loss of these sequences after divergence from the fish lineage 400-450 million years ago, or alternatively introduction of *texim* into the fish lineage through horizontal gene transfer.

**Figure 4:**
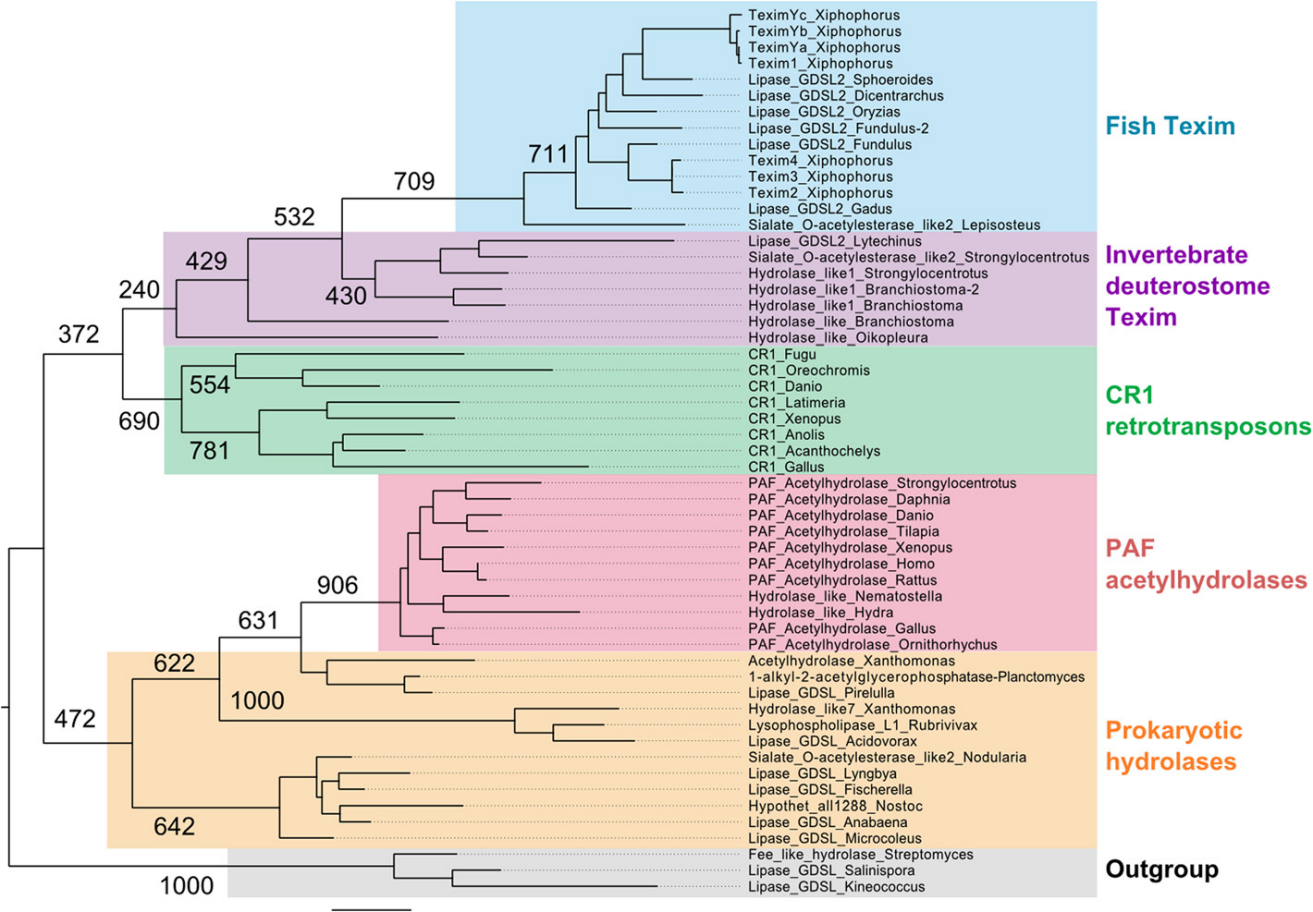
**Maximum likelihood phylogenetic tree of Texim-related SGNH hydrolase protein domains found in different organisms.** The Maximum Likelihood phylogenetic tree is based on an alignment of 112 amino-acids from the SGNH hydrolase domains. Bootstrap values are indicated (1000 repetitions). Accession numbers: Fish Texim: Lipase_GDSL2_Sphoeroides: *Sphoeroides nephelus*, protein prediction, AF094698.1; Lipase_GDSL2_Dicentrarchus: *Dicentrarchus labrax*, protein prediction, LG1, FQ310506.3; Lipase_GDSL2_Oryzias: *Oryzias latipes*, protein prediction, ENSEMBL, LG1:scaffold 395; Lipase_GDSL2_Fundulus-2: *Fundulus grandis*, JW608837.1; Lipase_GDSL2_Fundulus: *Fundulus grandis*, JW617431.1; Lipase_GDSL2_Gadus: *Gadus morhua*, consensus sequence from ESTs EL616197.1, ES481574.1 and ES474748; Sialate_O-acetylesterase_like2_Lepisosteus: *Lepisosteus oculatus*, protein prediction, LG7. Invertebrate deuterostome Texim: Hydrolase_like1_Amphioxus: *Branchiostoma floridae*, XP_002590490.1; Hydrolase_like1_Amphioxus-2: *Branchiostoma floridae*, protein prediction, scaffold 113; Hydrolase_like1_Strongylocentrotus: *Strongylocentrotus purpuratus*, protein prediction, scaffold 80934; Lipase_GDSL2_Lytechinus: *Lytechinus variegatus*, JI442514.1; Sialate_O-acetylesterase_like2_Strongylocentrotus: *Strongylocentrotus purpuratus*, XP_003726742.1; Hydrolase_like_Amphioxus: *Branchiostoma floridae*, XP_002586334.1; Hydrolase_like_Oikopleura: *Oikopleura dioica*, CBY22153.1. Accession numbers for PAF acetylhydrolases, prokaryotic hydrolases, CR1 retrotransposons and outgroups are available upon request.

All identified Texim sequences form a phylogenetic group distinct from other proteins with SGNH domains (Figure 4). Within the SGNH hydrolase family, Texim proteins are related to proteins from very different origins, including bacterial hydrolases, animal platelet-activating factor acetylhydrolases (PAFah) and proteins encoded by animal CR1 retrotransposons. No reverse transcriptase was found to be associated with any *texim* sequence, suggesting that *texim* is not part of a CR1 retrotransposon.

### *Texim* is associated with *Helitron* transposons in fish but not in other animals

**Figure 5:**
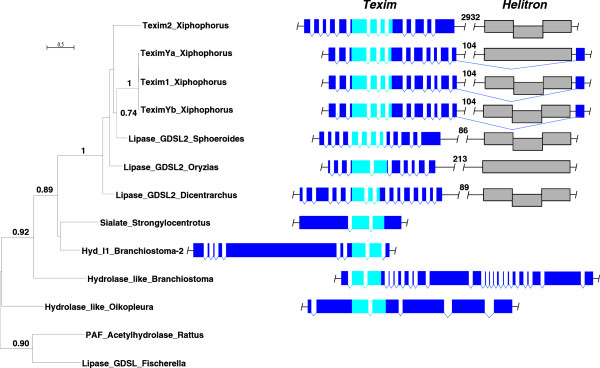
**Association of *texim *genes with *Helitron *transposons in different fish species, plotted on a Texim molecular phylogeny.***Texim* phylogeny was performed on a 112 amino-acid alignment using the Maximum Likelihood method with 100 bootstraps (values are provided). Blue boxes indicate *texim* exons, light blue regions encode the SGNH domain. Grey boxes show Helitron transposons, broken boxes indicate that the coding region is corrupted by point mutations. Nucleotide distance between *texim* and *Helitron* is provided. Accession numbers are given in the legend of Figure 4.

For all platyfish *texim* genes for which enough flanking sequence data was available for analysis, a *Helitron* transposon coding region was identified at the close vicinity 3′ from *texim* genes (3 kb downstream from the stop codon of *texim2*) or even in the last intron for *teximY/1* (Figures 2 and 5). *Helitrons* are eukaryotic rolling-circle DNA transposons [19,20]. They are able to capture and mobilize gene sequences in certain organisms and might therefore play a role in gene evolution through exon shuffling and gene duplication [21,22]. *Texim* was also found to be associated with *Helitrons* in other fish species including medaka, pufferfish and sea bass (Figure 5). Association between *texim* sequences and *Helitrons* transposons was found neither in *Oikopleura* (which does not have *Helitrons*) nor in Amphioxus and sea urchins (which contain *Helitrons*). These observations suggested capture and mobilization of an ancestral *texim* gene by a *Helitron* element in the fish lineage after divergence from urochordates/cephalochordates. “Free” *Helitron* transposons not associated with *texim* genes are present in zebrafish (which is devoid of *texim* genes) as well as in platyfish and other fish species possessing *texim-Helitron* associations.

### *TeximY* genes are preferentially expressed in testis in adult platyfish

**Figure 6:**
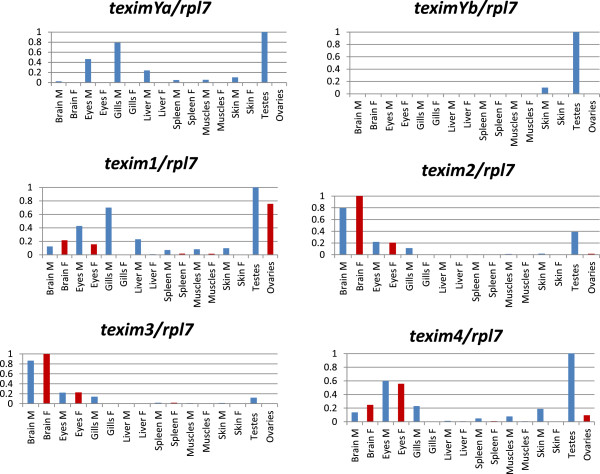
**RT-qPCR expression analysis of *texim *genes in adult tissues of *Xiphophorus maculatus *population Rio Jamapa.** All qPCR results were normalized against the ribosomal protein L7 housekeeping gene *rpl7*, the value 1 was assigned to the tissue/organ with the highest level of expression*.* M: males (in blue); F: females (in red). *TeximYa*, *teximYb*, *texim1* and *texim4* are preferentially expressed in testis, with *teximYb* being almost exclusively expressed in the male gonad. *Texim1* is also significantly expressed in ovaries. *Texim2* and *texim3* show predominant expression in brain.

**Figure 7:**
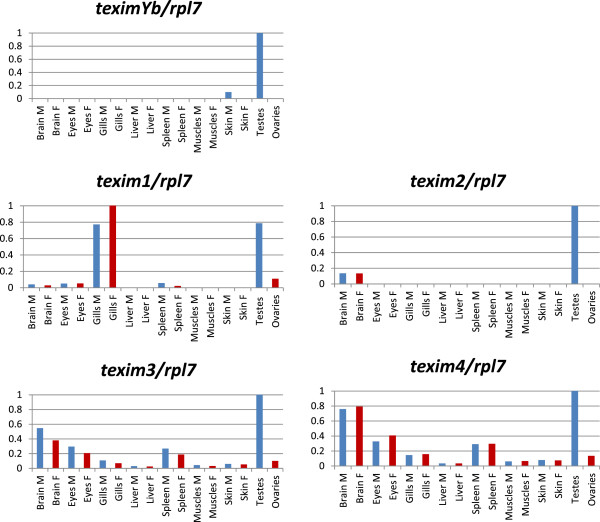
**RT-qPCR expression analysis of *texim *genes in adult tissues of *Xiphophorus maculatus *population Rio Usumacinta.** All qPCR results were normalized against the ribosomal protein L7 housekeeping gene *rpl7*, the value 1 was assigned to the tissue/organ with the highest level of expression*.* M: males (in blue); F: females (in red). All *texim* genes are strongly expressed in testis, with *teximYb* and *texim2* being almost exclusively expressed in the male gonad. *Texim1* is also strongly expressed in gills, and *texim4* in brain.

Expression of *X. maculatus texim* genes was analyzed by RT-qPCR in the Rio Jamapa population used to construct the BAC library (XY males and XX females, Figure 6), as well as in the Rio Usumacinta population (YY males and WY females) (Figure 7). The (pseudo)autosomal copies of *texim*, namely *texim1*, *texim2*, *texim3* and *texim4*, were all detected by PCR in genomic DNA of both males and females of the Rio Usumacinta population. From the three Y-linked copies, only *teximYb* was found in Rio Usumacinta. In this population, *teximYb* was detected by PCR in both males and females, suggesting localization either on the Y chromosome-specific segment or on (pseudo)autosomal regions.

In both populations, *teximYa* (when present) and *teximYb* were both preferentially expressed in testis, with *teximYb* being almost exclusively expressed in the male gonad (Figures 6 and 7). We could not detect any expression for *teximYc*, suggesting that this truncated sequence corresponds to a pseudogene. *Texim1* was strongly expressed in testis and also in gills in both platyfish populations. In addition, a significant expression was found in ovary in the Rio Jamapa population. Expression of other *texim* genes was generally strong in the testis, with exception of *texim2* and *texim3* in the Rio Jamapa population, which are predominantly expressed in the brain (Figure 6).

### *Texim* is expressed in spermatozeugmata and late spermatids in platyfish testis

**Figure 8:**
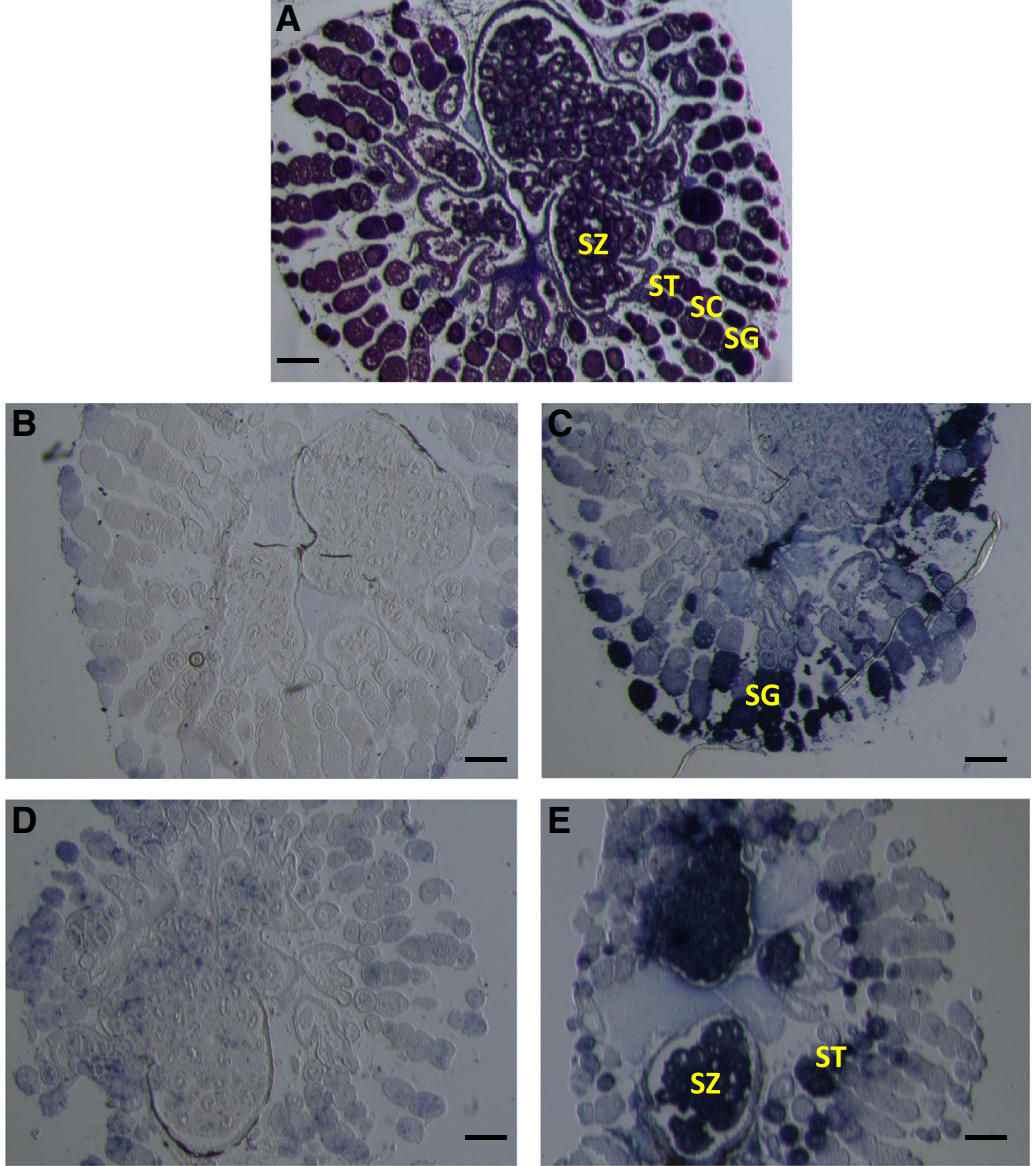
***In situ *RNA hybridization analysis of *vasa *and *texim1/Y *expression in platyfish adult testis. A**. Transversal testis section stained with Masson’s trichrome. **B**. *Vasa* sense probe, **C**. *Vasa* antisense probe, **D**. *Texim* sense probe, **E**. *Texim* antisense probe. SG, spermatogonia; SC, spermatocytes; ST, spermatids; SZ, spermatozeugmata. The probe used does not allow to differentiate between *teximY* and *texim1*. Scale bars: 50 μm.

Expression of *texim* gene in platyfish adult testis was further analyzed by *in situ* RNA hybridization. Histological analysis of the platyfish testis revealed the presence of many spermatozeugmata, i.e. the aggregation of mature sperm (Figure 8A). Hence, the testis analyzed was at stage VI of maturation (according to [23]). The *vasa* gene, encoding an RNA helicase of the DEAD box protein family, was used as a control for gene expression in germ line [24]. According to expression patterns observed in other fish species, *vasa* was expressed in spermatogonia and primary spermatocytes in the platyfish (Figure 8B/C). *In situ* hybridization for *texim1/teximY* (the probe used does not allow to differentiate between *teximY* and *texim1*) showed expression in late spermatids and spermatozeugmata, i.e. at late stages of spermatogenesis (Figure 8D/E).

## Discussion

Gene duplications play a major role in the formation of novel master sex-determining genes in fish [5,7,9] and other organisms [25,26]. New master genes appearing at the top of the sex determination cascade are generally derived from genes already involved in sexual development, as exemplified by the *dmrt1bY* master gene of the medaka [5,6]. However, exceptions may exist, as suggested for the immune gene paralog *sdY* recruited as the master sex-determining gene in the rainbow trout [9]. Frequently, master sex-determining genes in vertebrates encode transcription factors (*Sry* in therians, *Dm-W* in *Xenopus laevis*, *Dmrt1* in *Gallus gallus*, *dmrt1bY* in *Oryzias latipes*), but some recent data have demonstrated that it is not the general rule, at least in fish (*gsdf*^*Y*^ in *Oryzias luzonensis*, *amhY* in *Odontesthes hatcheri* and *amhr2 i*n *Takifugu rubripes*[7,8,10]). Importantly, all master sex-determining genes identified so far in fish are expressed in the same manner, in somatic cells surrounding the germ cells of the differentiating gonads.

In the platyfish *Xiphophorus maculatus*, an ovoviviparous teleost species, the master sex-determining gene has not been identified so far. Only a few functional genes have been described in the sex-determining region, including the multicopy melanocortin receptor gene *mc4r*, which controls the onset of puberty and is present on both the X and the Y chromosomes [14,27].

In this study, we have identified a new multicopy gene called *teximY*, triplicated on the Y but absent from the X chromosome. *TeximY* genes are located at distances between 80 and 280 kb near *Y-cript*, a Y-specific pseudogene linked to the sex-determining locus. The sex-determining region of the platyfish is very rich in repetitive DNA [16]. Accordingly, the repeat content of the 0.5 Mb region analyzed in this study is as high as 65.2% (unpublished results). In addition, the region containing the master sex-determining gene is characterized by a high level of genomic plasticity, with frequent rearrangements such as duplications, deletions and transpositions [12,16]. The presence of three copies of *texim* is consistent with the frequent duplications observed in this region. The autosomal *texim1* gene is a possible molecular ancestor of the Y-linked cluster. Y-chromosomal *texim* copies might be the result of mobilization by the flanking *Helitron* transposons, or might have been embedded in larger segmental duplication events. *TeximY* genes are preferentially expressed in the testis. Hence, the situation observed on the sex chromosomes of the platyfish is consistent with the observation that new sex-biased genes, especially male-biased genes, can be formed on sex chromosomes through the duplication of autosomal genes [28].

There is so far neither biochemical nor biological data concerning the functions of TeximY. Identification of conserved SGNH hydrolase domains in predicted sequences strongly suggests that Texim proteins work as esterases/lipases [17]. In contrast to master sex-determining genes described so far in fish, *teximY* genes are not expressed in somatic cells in the adult testis. Hence, *teximY* genes might not correspond to platyfish master sex-determining genes. However, additional expression analyses remain to be performed to test if these genes are expressed during embryonic development at the onset of sex determination.

We have shown that *teximY* genes are expressed in late spermatids and spermatozeugmata, suggesting that these genes might function at late stages of spermatogenesis. Interestingly, Texim is related to the platelet-activating factor (PAF) acetylhydrolase (PAFah) present in different animals. In mammals, PAFah is involved in reproduction [29-34], but also in other biological processes such as development, inflammation, hemostasis and apoptosis. This enzyme regulates PAF activity via deacetylation [29]. It reduces the level of PAF [33], but can also be involved in signaling pathways via interaction with other proteins in different tissues including testis [32,33,35,36]. Recently, a new testis-specific protein isoform of Pafah1b2 with possible role in spermatogenesis has been identified, the knock-out of which causes male infertility in the mouse [32].

Texim proteins are also related to prokaryotic proteins. Hence, either prokaryotic or eukaryotic *texim*-like genes share a very ancient common ancestor, or a horizontal transfer between prokaryotes and eukaryotes was involved in the evolution of *texim*-like sequences. In addition, Texim proteins show homology to proteins with esterase domains encoded by animal CR1 retrotransposons. These enzymes might play a role in interactions with cellular membranes, helping the element in penetrating host cells and possibly facilitating horizontal transfer [37]. *Texim* genes are not parts of retrotransposons, since they possess multiple introns and are not associated with reverse transcriptase genes. However, we cannot exclude that *texim s*equences have evolved from ancestral CR1-like elements.

Interestingly, *texim* sequences in fish are associated with another type of transposons, rolling circle DNA transposons called *Helitrons*. Such elements have been already identified on the sex chromosomes of the platyfish [38]. *Helitrons* are able to capture and mobilize resident coding sequences particularly in plants, and might therefore be involved in the evolution of gene functions through exon shuffling and gene duplication [21,22]. The absence of association between *texim* and *Helitrons* in urochordates, cephalochordates and echinoderms suggests that the capture event took place specifically in the fish lineage. *Texim* genes might facilitate *Helitron* transposition and transmission in a way similar to the role proposed for the Texim-related esterase in CR1 retrotransposons [37]. However, most *Helitron* transposons are not associated with *texim* in teleosts, indicating that mobilization and spread of *Helitrons* do not absolutely require Texim even in fish. Patchy distribution of *texim* in fish and other vertebrates suggests either recurrent loss of *texim* (e.g., in zebrafish and tetrapods) or horizontal transfer of *texim*, possibly in association with *Helitrons*.

## Conclusions

*Texim* genes in the platyfish uncover a novel multigene family encoding proteins with SGNH hydrolase domain, for which no functional data is available so far. The Y-specific copies *teximYa* and *teximYb* have no counterparts on the X chromosome and are specifically expressed in late spermatids and spermatozeugmata. *Texim* genes are associated with *Helitrons* in fish genomes, suggesting that they have been captured and mobilized by these transposons. Two non-exclusive hypotheses can explain these results. On the one hand, *texim* genes might have been maintained in *Helitrons* because Texim proteins facilitate the transposition or transmission of these elements. Alternately, *texim* genes might correspond to fertility genes with advantageous male functions mobilized and spread by *Helitrons* transposons. Such genes, which may have deleterious effects on females, are frequently located on the male-specific Y chromosome in animals. Fish present a very important evolutionary turn-over of sex chromosomes. Transposon-mediated mobility of fertility genes might allow the rapid fixation of spermatogenesis genes on neo-Y chromosomes, as suggested by the presence of three *teximY* genes on the Y but not on the X chromosome of the platyfish.

## Methods

### Fish

*Xiphophorus maculatus* males and females analyzed in this study belong to populations Rio Jamapa (Jp163A) and Rio Usumacinta (Up-2) reared at the Plateau de Recherche Expérimentale de Criblage *In vivo* (PRECI) of the SFR BioSciences Gerland - Lyon Sud (US8/UMS3444, Lyon, France) and the fish facility of the Biozentrum at the University of Würzburg (Germany). Fishes were kept under standard conditions and sacrificed following protocols in accordance with regulations from the French Ministry of Agriculture and the European Union (agreement number A693870602).

### *In silico* sequence analysis

Gene structure and protein sequences were predicted using FGENESH with the fish training set (http://softberry.com). Intron-exon structure was determined by sequence comparison with Expressed Sequence Tags (ESTs) downloaded from Genbank as well as by sequencing of RT-PCR and RACE-PCR products. BLAST analyses were mainly performed against sequence databases accessible from the NCBI (http://www.ncbi.nlm.nih.gov/BLAST/) and ENSEMBL (http://www.ensembl.org) servers. Sequence alignments were done using MUSCLE [39] via SeaView [40,41] and BioEdit [42]. Phylogenetic reconstructions were performed by Maximum Likelihood using PhyML 4.0 with 1000 bootstrap repetitions under the LG model, with the following additional parameters: optimized invariable sites, BioNJ as a starting tree and Best of NNI & SPR tree searching options [41]. The VISTA online software was used to perform alignments with mVISTA plot in order to visualize the level of sequence conservation (http://genome.lbl.gov/vista/mvista/submit.shtml). Conserved protein domains were detected via NCBI (http://www.ncbi.nlm.nih.gov/Structure/cdd/wrpsb.cgi), PFAM (http://pfam.sanger.ac.uk/) [43] and annotated with Gene Ontology terms (http://www.geneontology.org/) [44]. *Xiphophorus* autosomal and Y-linked *texim* sequences have been deposited in GenBank under accession numbers [KF433067- KF433073].

### Gene expression analysis

Total RNA was extracted from brain, eyes, gills, liver, spleen, muscle, skin and gonads of *X. maculatus* males and females from the Rio Jamapa and Rio Usumacinta populations using the TRIZOL reagent (Molecular Research Center, Inc.). Reverse transcription was performed with 1μg of total RNA using RevertAid First Strand Synthesis kit and oligo-dT primers (Fermentas). Expression patterns were determined by quantitative RT-PCR using gene-specific primer pairs. Primers used were GCTGCTTGCAGTTTGGATT and GTTTGATGGAGCCATGACA for *teximYa*, AGCCACGGGATGCAGAT and GTTCCTGTCCCTTAACCACAAC for *teximYb*, AGCCACGGGATGCAGAT and GTTCCTGTCCCTTAACCACAAC for *texim1*, CCTCTGCGGAAGAGTTGAAG and GTACGCAACAAAGCGTCAAA for *texim2*, GGAGACCTGCTGGACTCTTC and TTCCACCTTCGGACAACATA for *texim3*, and GACGCTCTGAGGCAGCTAA and CATCACGGGAGGCAACAT for *texim4*. Analysis of relative gene expression was done using the DeltaDeltaCt method [45]. All qPCR results were normalized against the ribosomal protein L7 housekeeping gene *rpl7* ([46,47] Additional file 1: Figure S1).

### *In situ* RNA hybridization

In order to generate probes for *in situ* hybridization, platyfish *vasa* (663 bp, [GenBank: KF424536], primers GGTTACCGTGGAAAAGACGA and CACCTTTCCTCTCCCAATCA) and *texim1* (542 bp, primers TTGGCTGTCTGTTCTCGTTG and GAGAAACGGACAGGATTGGA) partial cDNAs were amplified by PCR from testis. Amplified fragments were cloned into the pCR®II vector (Invitrogen Life Technologies). Digoxigenin (DIG)-labeled antisense RNA probes were synthesized by *in vitro* transcription from *Hin*dIII-linearized plasmid constructs using the SP6 RNA polymerase, while negative control sense RNA probes were produced from *Xho*I-linearized constructs using the T7 RNA polymerase. *In situ* hybridizations were performed on testis sections. Testes were fixed in 4% paraformaldehyde (PFA) in Phosphate Buffered Saline (PBS) buffer at 4°C overnight. After several washes in ethanol and then in toluene, testes were embedded in paraffin. Sections of 8μm were cut using a microtome and then slides were prepared. After deparaffinization of slides with xylene followed by several washes in 100% and 70% ethanol and PBS, testes were incubated for 20 min with proteinase K (20 μg/mL). Prehybridization was performed for 1 hour at room temperature in the hybridization buffer composed of 50% formamide, 20X Saline Sodium Citrate (SSC) buffer, 0.1% Tween-20, 10.5 μg/ml yeast tRNA, 0.05 mg/ml heparin, 5μg/ml salmon sperm DNA and 1X Denhardt’s solution in diethylpyrocarbonate (DEPC)-treated water. Both antisense and sense probes were diluted in the hybridization buffer for 3 min at 80°C. Slides with 100 μl of probe (1 μg/ml) were covered with hybridslips and hybridized in hybridization buffer at 70°C overnight. The next day, after removal of hybridslips in 5X SSC, slides were washed five times in hybridization washing solution (5X SSC, 50% formamide, 0.1% Tween-20 in distilled water) at 70°C for 30 min, then two times in 2X SSC at 70°C for 30 min and four times in Tris-Buffered Saline (TBS) buffer for 5 min. Slides were subsequently incubated for 1 hour in a blocking solution (1mg/ml, Roche), followed by incubation in an anti*-*DIG alkaline phosphatase conjugate (Roche). The next day, slides were washed four times in TBS for 5 min and two times in alkaline phosphatase buffer (NTMT; 0.1M Tris-HCl, 0.1M NaCl, 1M MgCl_2_, 0.1% Tween-20 in distilled water). Counterstaining was performed with a BM-purple solution (Roche) at room temperature until signal detection. Finally, slides were washed in PBS and mounted in Aquatex mounting media (Merck Milipore, Germany). Microphotographs were taken with Leica light microscope with 20X magnification.

## Competing interests

The authors declare that they have no competing interests.

## Authors’ contributions

MT performed molecular genetic experiments and bioinformatic sequence analyses, and drafted the manuscript. DC participated in bioinformatic sequence analyses. MS was involved in study design, interpretation of results and manuscript editing. DG contributed to the analysis of experimental results. JNV designed and coordinated the study, participated in data analysis and edited the manuscript. All authors read and approved the manuscript.

## Supplementary Material

Additional file 1: Figure S1 Basal expression profile of the *rpl7* gene in two populations of *Xiphophorus maculatus* Rio Jamapa and Rio Usumacinta. Two independent sets of cDNA have been generated from adult male and female organs: brain, eyes, gills, liver, spleen muscles, skin and gonads. Technical triplicates of each cDNA have been performed on the same qRT-PCR plate. Experiments were done with the Bio-Rad kit using the following PCR program: 40 cycles of 94°C and 59°C. Ct (Threshold cycle) values were averaged and standard deviation has been calculated. Click here for file
