# Supplementary figures and images for: A multicopy Y-chromosomal SGNH hydrolase gene expressed in the testis of the platyfish has been captured and mobilized by a Helitron transposon
Source: BMC Genet. 2014 Apr 8;15:44. doi: 10.1186/1471-2156-15-44 (PMC4021074; doi:10.1186/1471-2156-15-44)

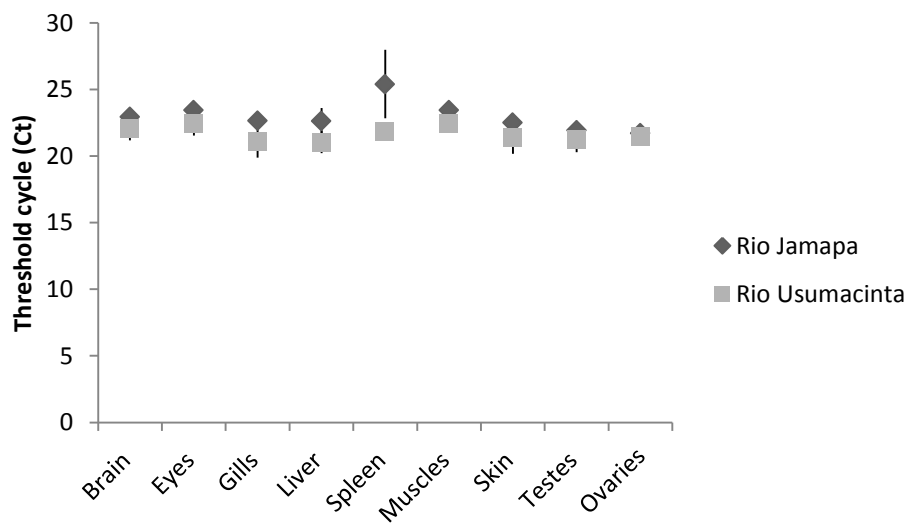

Supplement: Additional file 1: Figure S1 — Basal expression profile of the rpl7 gene in two populations of Xiphophorus maculatus Rio Jamapa and Rio Usumacinta. Two independent sets of cDNA have been generated from adult male and female organs: brain, eyes, gills, liver, spleen muscles, skin and gonads. Technical triplicates of each cDNA have been performed on the same qRT-PCR plate. Experiments were done with the Bio-Rad kit using the following PCR program: 40 cycles of 94°C and 59°C. Ct (Threshold cycle) values were averaged and standard deviation has been calculated. [file 1471-2156-15-44-S1.pdf]
